# Telling the truth to patients before hip fracture surgery

**DOI:** 10.1186/s12910-024-01067-3

**Published:** 2024-06-19

**Authors:** Rawan Masarwa, Merav Ben Natan, Yaron Berkovich

**Affiliations:** 1 The Orthopedics B Department, Hillel Yaffe Medical Center, Hadera, Israel; 2 Pat Matthews Academic School of Nursing, Hillel Yaffe Medical Center, P.O.B. 169, Hadera, 38100 Israel

**Keywords:** Telling the truth, Surgeon, Hip fracture

## Abstract

**Background:**

Hip fracture repair surgery carries a certain mortality risk, yet evidence suggests that orthopedic surgeons often refrain from discussing this issue with patients prior to surgery.

**Aim:**

This study aims to examine whether orthopedic surgeons raise the issue of one-year post-surgery mortality before hip fracture repair surgery and to explore factors influencing this decision.

**Method:**

The study employs a cross-sectional design, administering validated digital questionnaires to 150 orthopedic surgeons.

**Results:**

A minority of orthopedic surgeons reported always informing patients about the risk of mortality in the year following hip fracture surgery. The main reasons for not discussing this risk were a desire to avoid frightening patients, time constraints, and concerns about undermining patient hope. Orthopedic surgeons reported a medium-high level of perceived self-efficacy, with higher self-efficacy associated with a reduced likelihood of discussing one-year mortality risk. Conversely, older age and holding a specialist status in orthopedic surgery were associated with an increased likelihood of discussing this risk with patients.

**Conclusions:**

These findings suggest a need for interventions to address communication barriers and ensure consistent provision of essential information to patients undergoing hip fracture surgery. Additionally, they highlight the importance of considering individual factors such as self-efficacy, age, and expertise in designing strategies to improve patient-provider communication in orthopedic care settings.

**Trial registration:**

: The study doesn`t report the results of a health care intervention.

## Background

Hip fracture repair surgery is associated with improved patient survival rates [1], but also presents significant challenges during recovery, including a notable one-year mortality risk of approximately 20% [2–4]. Informed consent, a fundamental tenet of medical ethics, mandates that orthopedic surgeons communicate this risk to patients, as stipulated by the Patients’ Rights Law in Israel [5–7].

Despite these legal and ethical obligations, executing the informed consent process remains a challenge, especially in orthopedic surgery [8]. Malpractice claims often highlight inadequate disclosure of procedure risks [9], a deficiency attributed to various factors such as physicians’ aversion to delivering negative news, emotional discomfort, uncertainty regarding outcomes, and time constraints, particularly in urgent cases such as hip fractures [10, 11].

Physician attitudes towards disclosing unfavorable medical prognoses vary, as evidenced by a study in Israel where physicians, including surgeons, exhibited divided opinions on the matter [12, 13]. Emotional difficulty also influences physicians’ reluctance to convey bad news, as observed among orthopedic surgeons discussing mortality rates with hip fracture patients [10, 14]. Furthermore, uncertainty about patient comprehension or interest, compounded by the urgency of hip fracture surgeries, further complicates risk and prognosis discussions [6, 8].

Physicians’ communication skills also impact risk disclosure. Surgeons, often perceived as focused on the physical aspects of care, may overlook the emotional and psychosocial needs of patients [1, 15]. Deficiencies in communication, including inadequate assessment of patient understanding and lack of attention to emotional aspects, have been noted, highlighting the need for improved patient-centered communication practices [15, 16]. An Israeli study echoed these findings, revealing patients’ dissatisfaction with surgeons’ communication during the informed consent process [16]. To address the challenges outlined above and contribute to enhancing patient-provider communication in orthopedic surgery, the purpose of the present study is to explore whether orthopedic surgeons in Israel inform their patients of the mortality risk of hip fracture repair surgery, as well as possible reasons for informing or not informing.

## Methods

### Research design

The study was a cross-sectional survey.

#### Ethical approval

This study received approval from the Institutional Review Board (IRB) at the investigator’s institution.

### Sample

This study employed a convenience sample of 150 orthopedic surgeons. The inclusion criterion was being a specialist in orthopedic surgery or a resident. The decision to include both categories of participants was based on the understanding that both experienced surgeons and residents play vital roles in patient care and may encounter situations where discussions about one-year post-surgery mortality are relevant. Additionally, including both groups allows for a comprehensive examination of factors influencing communication practices across different levels of expertise within the orthopedic surgery specialty. The exclusion criterion was orthopedic surgeons and residents who had been working in the field for less than six months. The rationale for excluding orthopedic surgeons who had been working in the field for less than six months was to ensure a minimum level of experience and familiarity with orthopedic surgery practice. Residents who have recently entered the field may still be in their initial stages of training and may not have accumulated sufficient clinical experience to provide meaningful insights into the communication practices related to hip fracture repair surgery. By excluding this group, we aimed to focus on participants who have had a sufficient duration of exposure to orthopedic surgery practice to contribute relevant perspectives to our study.

### Instrument

The research questionnaire was comprised of 4 parts. The first part collected sociodemographic and professional information on the orthopedic surgeons (12 items). The second part was based on the Self-Efficacy in Patient Centeredness Questionnaire (SEPCQ-27) designed by Zachariae et al. [17] The original validation process of this questionnaire involved psychometric testing to establish its reliability and validity. This part examined orthopedic surgeons’ confidence in their communication skills during the informed consent process (23 items, e.g., ‘’I am confident in my ability to provide the patient with comprehensive information for informed consent to hip fracture repair surgery’’). Responses to these items were provided on a 5-point Likert scale, where 1 – strongly disagree, and 5 – strongly agree. A higher mean score means higher confidence. The internal reliability of this subsection was 0.80.

The third part of the questionnaire was designed by the researchers and examined orthopedic surgeons’ actions during the informed consent process prior to hip fracture repair surgery. Respondents were asked to rank the frequency with which they perform these actions on a 5-point Likert scale, where 1 – never, and 5 – always (3 items, e.g., ‘’I inform the patient regarding the mortality risk during the first year after the surgery’’). The internal reliability of this subsection was 0.72.

The fourth part of the questionnaire was designed by the researchers and examined reasons for not informing the patient regarding one-year mortality risk following the hip fracture repair surgery (7 items, e.g., ‘Lack of time’). Respondents were asked to rank their degree of agreement that the different factors represented reasons for not informing the patient, on a 5-point Likert scale, where 1 – strongly disagree, and 5 – strongly agree. The internal reliability of this subsection in the present study was 0.82.

We enhanced the questionnaire’s validity by engaging three content experts in orthopedic surgery, who provided valuable insights and feedback. Their expertise ensured the relevance, clarity, and appropriateness of the questionnaire items for our study population. Although we did not conduct a formal psychometric validation within our specific sample, the thorough review process with these experts ensured the content validity of the questionnaire. As a result of their input, several items were modified to maximize the clarity and comprehensibility of the questionnaire. While not explicitly labeled as a Delphi process in our methodology, our iterative review process with content experts shares similarities with aspects of the Delphi method. This approach allowed for consensus-building and refinement of the questionnaire items through multiple rounds of feedback and revision.

### Procedure

After receiving approval by the IRB at the investigator’s institution, a list of e-mail addresses of orthopedic surgeons in Israel was obtained. Subsequently, a link to the online questionnaire was distributed via e-mail to potential participants. The questionnaire was accompanied by an explanation of the purpose of the study. Respondents were informed that participation in the study was voluntary and anonymous. They were then asked to provide their e-consent to participation through a specific dichotomous question (i.e., Yes vs. No). No incentives were provided to the research participants. The questionnaire was sent to 200 orthopedic surgeons and 150 questionnaires were returned completed, for a response rate of 75%.

### Statistical analysis

The data were analyzed using the SPSS for Windows (version 27.0, SPSS Inc., Chicago, IL, USA) statistical software package. Means, standard deviations, and percentages were used to describe the sample’s characteristics. T-tests for independent samples and a Chi-square test were employed to identify differences between informers and non-informers. A linear regression was performed to identify predictors of informing the patient about mortality risk. A p-value of < 0.05 was considered statistically significant.

## Results

The research participants consisted of *N* = 150 orthopedic surgeons, with a mean age of M = 44.43 ± 10.68 and an age range of 27–73. 80% (*n* = 120) of the surgeons were men. Most were married (73.2%, *n* = 109), where their mean number of children was M = 2.0 ± 0.69 and a range of 1–7 children. Eight of the orthopedic surgeons had no children. Of all surgeons, 68.7% (*n* = 103) were Jewish and 31.3% (*n* = 47) Arab. Most were secular (61.3%, *n* = 92). 62% (*n* = 93) had studied in Israel and 38% (*n* = 57) elsewhere. The percentage of specialists in orthopedic surgery was 69.8% (*n* = 104), while 30.2% (*n* = 46) were residents. The mean number of years as a physician was M = 14.56 ± 9.49 and the mean number of years as a specialist in orthopedic surgery was M = 10.02 ± 8.07. 74% (*n* = 108) said that they had received training on informed consent, while 26% (*n* = 42) said that they had not. On average, the surgeons in this study performed *N* = 31.12 ± 3.07 hip fracture surgeries in the past year, with a range of 1-150 surgeries (Table 1).

**Table 1:** Sociodemographic details of the research population (*N* = 150)

		*n*	%	M	SD
Gender	Male	120	80%		
	Female	30	20%		
Marital status	Single	25	16.8%		
	Married	109	73.2%		
	Divorced	11	7.4%		
	Separated	2	1.3%		
	Widowed	2	1.3%		
Number of children				2	1.1
Sector	Jewish	103	68.7%		
	Arab	47	31.3%		
Religiosity	Secular	92	61.3%		
	Traditional	49	32.7%		
	Religious	7	4.7%		
	Ultra-orthodox	2	1.3%		
Location of medical studies	Israel	93	62%		
	Abroad	57	38%		
Level of specialization	Expert	104	69.3%		
	Resident	46	30.7%		
Length of experience				14.56	9.4
Training on informed consent		108	72%		
Number of hip fracture surgeries performed in the past year				31.12	3.07

When examining the perceived self-efficacy of communication with patients before surgery for hip fracture repair, it is evident that the mean level of self-efficacy was medium-high (M = 3.92 ± 0.7). Thus, 75% of orthopedic surgeons claimed that they are certain of their ability to inform the patient of anticipated side effects and to verify that the patient understands them. In addition, 72.7% claimed that they are certain of their ability to provide a full explanation of informed consent before hip fracture surgery and 74.5% claimed that they are certain of their ability to separate their personal views from the professional care. Furthermore, 72% claimed that they are certain of their ability to consult and support patients in reaching care-related decisions. In contrast, only 2.7% (*n* = 4) reported that they always inform the patients that there is a risk of mortality in the year after a hip fracture surgery, and only 5.4% (*n* = 8) reported that they always inform the patients that there is a high risk of deterioration in their functional state post-surgery.

The research sample was divided into two groups: orthopedic surgeons who often or always inform patients of the risk of mortality (henceforth: “informers”) and orthopedic surgeons who rarely inform or do not inform (“non-informers”). Among non-informers, the prevalent reasons for the decision to refrain from informing patients of the risk of mortality in the year after surgery were the wish to avoid frightening the patient (45%), the concern of causing patients to lose hope (39%), and lack of time (39%).

A significant difference was found between informers and non-informers in their perceived self-efficacy and ability to communicate with patients when providing guidance before hip fracture repair surgery [t=-2.43 (df = 148), *p* < 0.02]. Thus, non-informers had higher self-efficacy (M = 3.92 ± 0.66) than informers (M = 3.02 ± 0.56). In addition, informers were also found to be older on average, with a greater length of experience as physicians and a greater length of experience as specialists in orthopedic surgery (Table 2).

**Table 2:** Differences in the characteristics of the groups: Informers versus non-informers of the risk of mortality in the year after hip fracture repair surgery

	Informers		Non-informers		t	df
	SD	M	SD	M		
Age	0.6	38.72	0.1	45.01	-1.98	147
Length of experience as physician	0.68	9.09	0.05	15.00	-2.00	147
Length of experience as expert	0.42	5.6	0.8	11.35	-1.96	104

Table 3 presents the results of logistic regression analysis aimed at predicting the likelihood that orthopedic surgeons would inform patients of the risk of mortality in the first year after hip fracture surgery. Our analysis revealed that older age and a specialist status were significant predictors associated with an increased likelihood of orthopedic surgeons informing patients about the risk of mortality post-surgery. Specifically, for every one-unit increase in age, there was a 1.82 times higher odds of surgeons informing patients about mortality risk, and surgeons classified as specialists in orthopedic surgery were 2.25 times more likely to discuss this risk compared to residents.

**Table 3:** Logistic regression for predicting informing the patient of the risk of mortality after hip fracture repair surgery

	OR (95% CI)	*P*-value
Age	1.82 (1.04–3.18)	0.04*
Gender (women men)	1.02 (0.92–1.06)	0.378
Marital status	0.73 (0.27–2.06)	0.556
Religiosity	0.90 (0.52–1.06)	0.478
Expert level (expert or resident)	2.25 (1.31–3.89)	0.02*
Self-efficacy	0.67 (0.20 to 0.92)	0.03*

Conversely, higher levels of self-efficacy were found to decrease the likelihood of surgeons informing patients about mortality risk, with a one-unit increase in self-efficacy associated with a 0.67 times lower odds of discussing this risk. Notably, variables such as gender, marital status, and religiosity did not show significant associations with the likelihood of surgeons informing patients about mortality risk.

## Discussion

The current study examined whether orthopedic surgeons raise the issue of one-year post-surgery mortality before hip fracture repair surgery, and the reasons for informing or not informing the patient. The research results indicate that only a minority of orthopedic surgeons always inform the patients of the risk of mortality in the year following a hip fracture surgery. In addition, only a small number always inform the patients of the risk of post-surgery deterioration in their functional state. These findings are consistent with previous studies showing that orthopedic surgeons fail to discuss the risks and prognosis of orthopedic procedures [2, 5]. The findings are also consistent with a previous Israeli study that revealed that half the Israeli physicians object to disclosing the whole truth about a poor medical prognosis, while the attitude of surgeons did not differ from that of other specialists [18].

This conduct by orthopedic surgeons seems to circumvent to a certain degree their obligations according to the Patients’ Rights Law, which states that medical treatment shall not be administered to a patient unless he/she has given his/her consent after being provided with all the necessary information, including the risks of the procedure [7]. Namely, the orthopedic surgeons in the current study appear to have asked the patients to sign the informed consent form without providing them with the full information. Moreover, the Israeli Patients’ Rights Law states that physicians may withhold medical information that, in their opinion, may cause serious harm to the patient’s health or endanger his/her life. However, such refusal requires the approval of an Ethics Committee [7]. In the current study, it appears that the orthopedic surgeons decided at their discretion to leave out information regarding the risk of mortality.

In our study, we identified several key factors contributing to orthopedic surgeons’ decisions to withhold information regarding the risk of mortality in the year following hip fracture repair surgery. These included the wish to avoid frightening the patient, concern of causing patients to lose hope, and lack of time, in consistence with the literature [2, 6, 10].

Notably, among orthopedic surgeons who opted not to inform patients of the mortality risk, the predominant reasons were closely tied to patient-related considerations, in except of time constraints, attributable to systemic issues rather than solely reflecting individual surgeon-related factors. These findings highlight the complex interplay between patient-related factors, such as emotional well-being and understanding, and systemic constraints within the healthcare environment. Such insights underscore the importance of tailored communication approaches that prioritize patient-centered care while addressing structural barriers that may impede comprehensive discussions about mortality risks and prognosis in orthopedic settings.

Our study found that orthopedic surgeons reported a medium-high level of perceived self-efficacy in communicating with patients, yet higher self-efficacy was associated with a reduced likelihood of discussing one-year mortality risk. This seemingly contradictory finding suggests that while surgeons may feel confident in their ability to communicate, they may also exercise discretion in selectively disclosing information based on their own judgment and perception of patient needs.

Moreover, the decision to disclose mortality statistics may be influenced by ethical considerations, including concerns about patient autonomy and the principle of beneficence. Orthopedic surgeons may grapple with the delicate balance between providing patients with comprehensive information to make informed decisions and protecting them from unnecessary distress or anxiety.

Overall, the reluctance of orthopedic surgeons to disclose mortality statistics during the informed consent process reflects a complex interplay of patient-centered and surgeon-centered factors. Further research is needed to explore these dynamics in greater depth and develop interventions to support more transparent and patient-centered communication practices in orthopedic care settings.

In the current study, the orthopedic surgeons reported a medium-high level of perceived self-efficacy and ability to communicate with patients while providing guidance before hip fracture repair surgery. These findings are not consistent with previous studies that report deficiencies in communication between surgeons and their patients during the informed consent process [15, 16]. The current findings show that orthopedic surgeons may perceive themselves differently than their perception by others. Nevertheless, it is notable that the current study explored orthopedic surgeons’ perception of their efficacy rather than their actual behavior. Namely, orthopedic surgeons may have an exaggerated perception of their abilities that does not necessarily reflect reality. Among other things, they may be so certain of their past behaviors based on emulating specialists that they feel they have high self-efficacy.

The current study found that non-informing orthopedic surgeons had higher perceived self-efficacy than informing surgeons. Moreover, higher self-efficacy was found to reduce the likelihood that the orthopedic surgeon would inform the patient of the risk of mortality in the year after hip fracture surgery. This finding seems to encompass a certain contradiction, as the expectation is that a surgeon with higher self-efficacy for communicating with patients will also know how to convey to patients less attractive information. However, high self-efficacy may reflect humane sensitivity that might cause the surgeon to refrain from conveying all the information. In contrast, orthopedic surgeons with low perceived self-efficacy may be “insensitive” and therefore have no problem telling the patient the whole truth. But once again, this hypothesis is less likely if the perceived efficacy does not reflect the actual circumstances.

The current study also found that older age raises the likelihood that the orthopedic surgeon will inform the patient of the risk of mortality in the year after a hip fracture surgery. Moreover, in the current study also the status of a specialist in orthopedic surgery was found to raise the likelihood that the orthopedic surgeon would inform the patient of the risk of mortality in the year after a hip fracture surgery. These findings are not consistent with a previous Israeli study where support for disclosing the whole truth about a poor medical prognosis was higher among younger physicians, while professional experience was not associated with this attitude [12]. The inconsistency between the previous and current findings may be associated with the difference between the research populations and the specificity of the issue of telling patients the whole truth. Thus, in the previous study the physicians were asked to state their attitude to telling the truth in general, while in the current study the orthopedic surgeons were asked about telling the truth regarding a specific issue, namely informing patients about the risk of mortality. One potential explanation for the findings in our study is that older orthopedic surgeons with expert status may be more inclined to discuss the risk of mortality with patients due to their accumulated experience in similar scenarios. Additionally, it is plausible that other unmeasured factors, such as burnout, could contribute to a decreased sensitivity towards disclosing the whole truth to patients. While burnout may be a relevant consideration, it is important to note that our study did not directly assess or measure burnout among orthopedic surgeons.

Future research could explore the applicability of the questionnaire across various surgical specialties to assess its comprehensiveness in capturing key aspects of the informed consent process. Piloting the questionnaire in different clinical settings and specialties would provide valuable insights into its relevance and effectiveness in facilitating patient-provider communication and shared decision-making.

### Research limitations

The current study has several limitations. Thus, since the research findings are based on a convenience sample, there is a limited ability to generalize from the findings, particularly in light of the 75% response rate. In addition, due to the sensitivity of the topic, the research findings might have been influenced by social bias. Moreover, the research design – a cross-sectional study – does not make it possible to determine causality.

## Conclusions and recommendations

The current findings indicate that only few orthopedic surgeons make a point of informing patients about the risk of mortality in the year after a hip fracture surgery and the high risk of deterioration in one’s functional state after surgery, conduct that circumvents the Patients’ Rights Law to a certain degree. It was also evident that this probably stems from similar motives as those of other physicians, i.e., a wish to refrain from frightening the patient, lack of time, and concern of causing patients to lose hope. This conduct too was found to be more typical of older orthopedic surgeons with a greater length of experience.

Moreover, the research findings suggest a certain association between perceived self-efficacy and the ability to communicate with patients before hip fracture repair surgery, particularly in conveying less favorable information. Surprisingly, higher self-efficacy was associated with a reduced likelihood of informing patients about the risk of mortality. However, it’s important to note that self-efficacy alone may not fully capture competency, especially among residents who may be early in their training. Given this consideration, future studies could explore the impact of standardized training protocols, ensuring both residents and specialists receive consistent briefing in obtaining consent and disseminating information. Further research is warranted to corroborate these findings and explore the role of training programs in enhancing communication practices among orthopedic surgeons.

In summary, the practices of orthopedic surgeons regarding the disclosure of less favorable aspects of surgery appear to align with those of other physicians. This behavior is influenced by a variety of factors, including personal attributes, professional norms, and organizational dynamics. It is therefore recommended that initiatives be undertaken to enhance awareness among orthopedic surgeons regarding the importance of addressing such issues with patients. Furthermore, future research endeavors should consider integrating patient-reported outcome measures to comprehensively capture their perspectives and levels of satisfaction.

Moreover, there is a crucial need for further investigation into the intricate interplay between physician characteristics, organizational environments, and patient communication practices. This exploration should encompass a broad spectrum of factors, including but not limited to the potential impact of burnout on physician behavior. By delving deeper into these dynamics, future studies can contribute to a more nuanced understanding of the complexities surrounding patient-provider interactions in orthopedic care settings.

## Data Availability

The dataset used and analysed during the current study is available from the corresponding author on reasonable request.
